# Precise Temperature Measurement for Increasing the Survival of Newborn Babies in Incubator Environments

**DOI:** 10.3390/s141223563

**Published:** 2014-12-08

**Authors:** Robert Frischer, Marek Penhaker, Ondrej Krejcar, Marian Kacerovsky, Ali Selamat

**Affiliations:** 1 Department of Cybernetics and Biomedical Engineering, Faculty of Electrical Engineering and Computer Science, VSB-Technical University of Ostrava, 17. Listopadu 15, Ostrava Poruba 70833, Czech Republic; E-Mails: Robert.Frischer@vsb.cz (R.F.); Marek.Penhaker@vsb.cz (M.P.); 2 Center for Basic and Applied Research, Faculty of Informatics and Management, University of Hradec Kralove, Rokitanskeho 62, Hradec Kralove 50003, Czech Republic; 3 Biomedical Research Center, University Hospital Hradec Kralove, Hradec Kralove 50003, Czech Republic; E-Mail: Marian.Kacerovsky@fnhk.cz; 4 Faculty of Computing, Universiti Teknologi Malaysia and UTM-RDA Center of Excellence, UTM Johor Bahru, Johor 81310, Malaysia; E-Mail: aselamat@utm.my

**Keywords:** temperature measurement, incubator, sensitivity increasing, A/D converter

## Abstract

Precise temperature measurement is essential in a wide range of applications in the medical environment, however the regarding the problem of temperature measurement inside a simple incubator, neither a simple nor a low cost solution have been proposed yet. Given that standard temperature sensors don't satisfy the necessary expectations, the problem is not measuring temperature, but rather achieving the desired sensitivity. In response, this paper introduces a novel hardware design as well as the implementation that increases measurement sensitivity in defined temperature intervals at low cost.

## 1. Introduction

Considering the measurement of physical parameters is mostly applicable in industrial and medical environments, a significant emphasis is placed on potential health risk for patients, particularly in the medical field. Although measuring and testing in incubators for newborn babies are practical, these applications lead to the problem with the need for a very precise temperature measurement inside the incubator. If the measurement and following temperature control fail, a newborn baby could die in a few minutes [1,2].

Preterm delivery (PTD) which is defined by the World Health Organization as delivery occurring at less than 37 gestational weeks or before 259 days [3], is the primary cause of perinatal mortality and associated with up to 75% of long-term perinatal morbidity, such as cerebral palsy, developmental delay, retinopathy of prematurity, and other conditions. Despite progress in perinatal medicine, knowledge about risk factors and mechanisms related to this pregnancy complication, PTD rates are still between 5% and 9% in Europe and in other developed countries, while in the USA, this rate was increased to 12.7% in 2005 [4–6].

Premature newborns need extra monitoring, treatment and care since they are more vulnerable than the newborns delivered on the expected date and can have serious health problems. One of the most common features of the care for premature newborns is the usage of incubators that provide stable, appropriate temperature and humidity. This has substantial importance since heat production requires oxygen consumption and usage of glucose. Moreover, persistent hypothermia may lead to metabolic acidosis, hypoglycemia, decreased surfactant production, increased caloric requirements, and if chronic, impaired weight gain.

In addition to premature newborns, incubators are also used for the following purposes: for newborns who are too small for their gestational age as well as infants unable to maintain their own temperature with clothing and wrapping, risk of abnormal heat loss, nutritional problems, large wounds or infection.

Given the problem of temperature measurement, although there are many sensors which can measure temperature, each has advantages and disadvantages. These sensors transform temperature into resistance or voltage, but measurement accuracy is always a main concern and objective. When designing a device it is necessary to obtain accurate temperature readings and to select a proper sensor type. Resistive-based sensors such as thermistors are relatively cheaper and temperature can be retrieved simply. On the other hand, accuracy is thus lower and each sensor should be calibrated to obtain significant data. Voltage-based sensors (thermocouples) are much more precise, nonetheless, since output voltage is very low (a few mV), and amplification errors might be very high. Low offset, precise operation amplifiers would be mandatory, as these types of sensors are preferred. In this paper a standard LM35 resistance based sensor is chosen, yet the main disadvantage is the low sensitivity when measuring human body temperatures. It certainly is possible to use precise operation amplifiers, however it would be necessary to use an A/D converter with higher resolution. All these conditions point to high overall complexity and high costs. Basically, the concern of this paper is to propose a simple, low cost sensor for measuring the temperature of a newborn baby within reasonable accuracy. As the standard approach to this issue cannot be used, we furthermore developed our novel solution based on the precise measurement area with aspects of electrical engineering. Finally we describe the idea of the solution as well as the design, the hardware implementation and the results that are given at the end of the article.

## 2. Problem Definition

**Figure 1.**
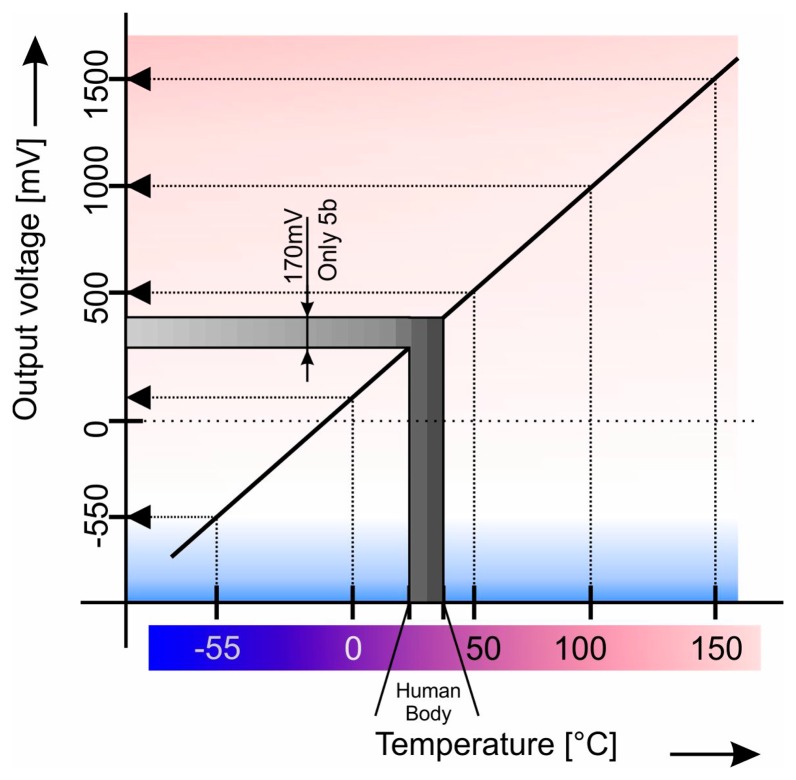
Human body temperature range *vs.* sensor measuring range.

As mentioned before, a solution to the problem of measuring a certain quantity in a short range with reasonable accuracy is not achievable. For instance, when measuring the temperature range from 0 °C to 200 °C with 10 b A/D, the maximum resolution would be at 0.2 °C assuming full coverage of signal voltage. As such, the aspect ratio between temperature range and accuracy becomes 1000:1 which is a fair value. If we use the same circuit to measure a temperature range from 35 °C to 45 °C the final accuracy would be the same and aspect ratio between temperature range and accuracy will however be only 50:1, which is actually a poor value so uncertainty is very high due to the utilization of a small range, as can be seen in Figure 1. Ideally, it is necessary to cover all ranges of the A/D converter in the temperature range that is required.

## 3. Related Work

Several existing solutions are presented in the literature for the precise temperature measurement problem studied in this paper. Chang *et al.* [7] proposed a method to measure the temperature precisely by A/D and therefore adapted the circuit to higher demands. They also created a precise voltage reference to use the thermal dependence of NMOSFET semiconductor devices. This step is mandatory, since resistance differences in these types of devices is very small and sensitive to any discontinuities. As the paper uses a standard OP amp interconnection to amplify temperature data the temperature range is from −20 °C to 120 °C and accuracy is about 0.55 °C/LSB though this implementation is inapplicable to human body temperature reading.

Additionally, Huang *et al.* [8] introduced a method of air temperature measurement by changing the air features along with the temperature change. More precisely, this method focused on changes of the sound speed in air dependent upon temperature adaptation. This method calculates the time between outbound signal pulses and inbound responses which depends on temperature, if the distance between the transmitter and receiver is constant. This unusual method is relatively precise, with an absolute measurement error of about 0.2 °C and can be used for measuring the temperature in infant incubators.

Although the speed of sound in air is also dependent on relative humidity this paper, however didn't involve this dependence in the final formula. Therefore, the total error of the presented method will obviously be slightly higher than the presented ±0.2 °C, due to humidity fluctuations over time.

Finally, Elsgaard and Jorgensen [9] made a special temperature-gradient incubator (TGI) with highly precise temperature regulation. This article describes a modern approach to temperature regulation using Peltier elements which seems very accurate at keeping the temperature in small volumes at a constant value. Peltier modules are easy to drive, since they are mainly semiconductor diodes connected in series—parallel manner. As the current passes through, the temperature gradient appears on opposite sides. Additionally, when using Peltier cells the temperature error, can be as low as 0.008 °C. An excess demand emerges on temperature sensors due to the temperature gradient in which the sensors are placed. The authors achieved a measuring accuracy of 0.02 °C using a special, highly accurate and expensive Tempmaster-100 thermometer and Pt-100 temperature sensors which are highly precise and expensive resistance-based ones. Given these results, summarized the Table 1, it is necessary to develop new solutions to meet all requirements since it is crucial to take the other works compared one step further.

## 4. Proposed Solution

This section introduces the development of a new device to increase the desired sensitivity while measuring temperatures between the 25 °C and 42 °C in medical environments. There are plenty of analog sensors in the market, however, their overall accuracy is also dependent on the input A/D converter. Even though it is possible to buy an expensive, precise sensor on the market, the measurement error would be relatively high if the A/D converter is only 8 b or 10 b. The sensitivity of the sensor is calculated by the following Equation (1):

(1) $${\text{Sensitivity}}_{\pm \text{LSB}}=\frac{U_{\text{ref}}}{2^{n}}$$

where *U_ref_* is reference voltage level of A/D, usually about 5 V and *n* is resolution (length of single converted data). If 10 b A/D is used, the sensitivity would be 4.88 mV which seems to be a sufficient value, however, if the temperature is represented by a sensor having 10 mV/°C, then we can measure temperature with an accuracy of around 0.5 °C, which is far from a satisfactory level. Another issue which needs to be considered is that human body temperature varies from 25 °C to 42 °C therefore there is only a 17 °C difference to be measured. If the sensitivity is 0.5 °C, only 34 values would be obtained, consequently, which corresponds to a 5 b A/D converter. This is an excessive wasting of time in the measurement hardware that is used for measuring temperature and in order to avoid this problem, another temperature sensor with better sensitivity and better A/D converter with 16 b or 24 b accuracy could be used. When using a 24 b A/D converter, the 17 °C scale is covered by 570,425 values which is equivalent to a 19 b A/D converter without any hardware modification. Given these facts it still seems sufficient, yet the costs and complexity of the whole solution would be very high.

Considering the facts mentioned above, a hardware modification and adaptation are recommended. The proposed solution is based on a novel hardware magnifier, which enables modifying the input temperature signal and adapting it to the traditional A/D converter.

## 5. Hardware Implementation

One way of increasing sensitivity is to use a more complex A/D converter, however, the overall complexity and price would not be as appropriate as a new solution. Moreover, a lower reference voltage could be used for A/D, which would increase the sensitivity yet it would not be significantly beneficial for this case. Furthermore, another way is to utilize a hardware “magnifier”. However the desired temperature range is very low as demonstrated in Figure 1. In comparison to the whole scale (5 V), the resulting voltage is only 170 mV however, and ideally, the reference voltage for A/D should vary from 250 mV to 420 mV which is very hard to reach.

**Figure 2.**
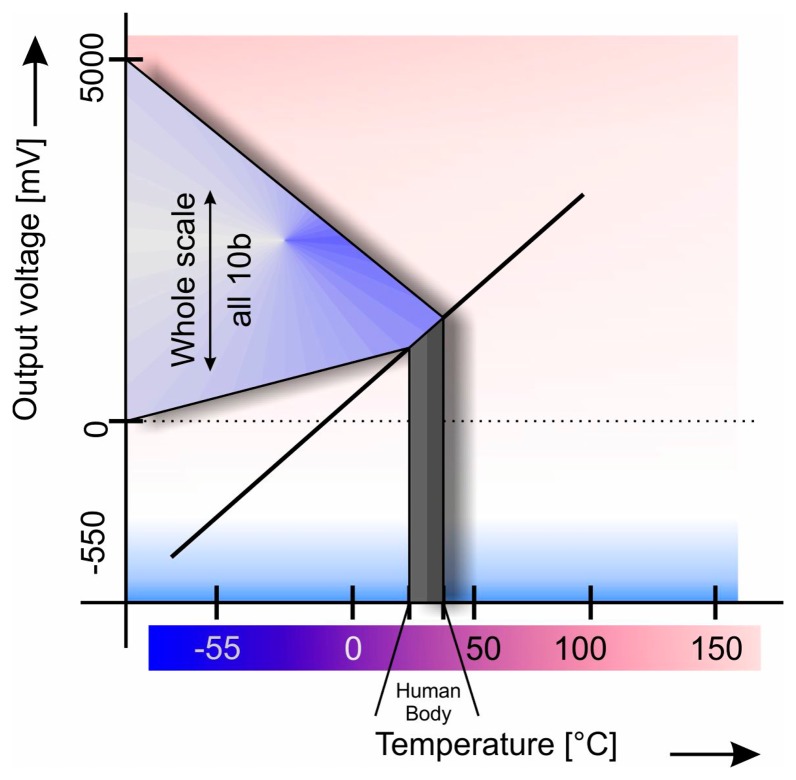
Small signal is magnified to full converter's scale to achieve maximum A/D resolution.

Even if given A/D converter provides a single solution that obviously wouldn't be a sound approach, therefore, changes have to be made from the sensor side. It is necessary to magnify a small voltage difference to full scale range—0 V∼5 V—as shown in Figure 2 and in order to achieve this, a hardware magnifier was constructed which enables us to enlarge any voltage difference. Not only voltage level relative to the ground level, but also the floating level which is important for our case, as the measurement range is shifted or restricted.

Mainly, the device consists of three parts; differential op amp with level shifter, negative power supply and output stage which is made of non-inverting operational amplifier with variable gain.

**Figure 3.**
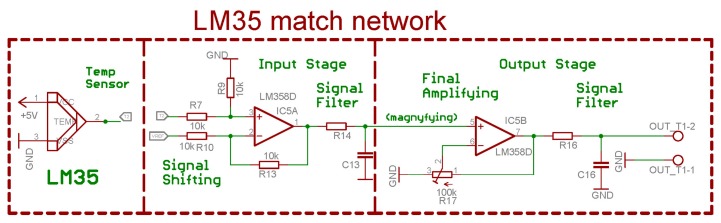
Input signal stage with basic signal adjustments.

The input stage consists of op amp (LM358), resistors (R7, R9, R10, R13) and output filter (R14 + C13) as can be seen in Figure 3. The gain of the input stage is set to 1, which is obvious since all of the resistors are the same, therefore it is only crucial to follow the measured difference and convert it to a simple signal relative to the ground signal. The shift of the reference voltage connected to R10 results in increasing or decreasing voltage therefore, it is possible to set the voltage level corresponding to a temperature of 25 °C as 0 V. The output voltage swings identically as the source, but relative to the permanent ground which is not floating and the output filter is only complementary. The temperature measurement process is a slowly changing system, which also means that no rapid transients are expected. This filter removes spikes and disturbances which leak from the supply voltage. Its values are not critical and depend on current conditions. The operational amplifier LM358, which is a standard low cost amplifier for low frequency applications was selected for further analysis. Due to the kernel of thermal systems, the temperature drift is usually not so dynamic.

**Figure 4.**
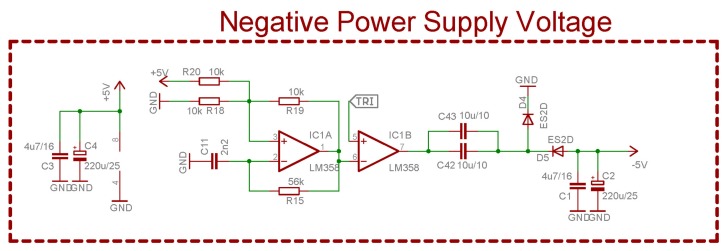
Negative voltage power supply—charge pump.

One main disadvantage of this op amp is its inability to deliver signals at a full range (0–5 V) or in other words, it is not a Rail-To-Rail device. Even if it had been such, voltage levels near the ground or supply voltage would be inaccessible. The supply voltage could be raised above 5 V to easily achieve the maximum voltage level of 5 V. In order to touch the ground level, it is necessary to use a negative power supply. Although one or two volts are enough under standard conditions however the negative supply is not accessible. Zero voltage is very important, since it corresponds to 25 °C therefore, a simple negative power supply was designed in Figure 4 which consists of IC1 A and IC1B, resistors (R15, R18, R19, R20), capacitors (C1, C2, C11, C42, C43) and two diodes (D4 and D5).

IC1 and the surrounding components make up a simple square generator of about 6 kHz while the final frequency can be tuned by R15 and C11. The output of this block is a square voltage pattern covering levels from 0 V to the approximate supply voltage. IC1B is only a power stage boosting output current which also copies input voltage from negative inputs. Capacitors C42 and C43 are in a parallel to double the capacity and to half the inner resistance (**R_ESR_**). If the output of the IC1B is at a high state, capacitors are charging themselves through D4 where if the output changes, one side of the capacitors is connected to the ground (pin 7, IC1B), yet this side was previously charged positively. The second capacitor's terminal would be now under the ground level which is lowered by voltage at which capacitors were charged. If a negative voltage level appears, capacitors C1 and C2 are charging through D5. As a result of this process, a negative voltage level is obtained on these capacitors therefore this voltage supplies other op amps and creates sufficient difference to represent 0 V levels at their outputs.

Finally, the signal from the temperature sensor is shifted, however the overall voltage swing is constant at 170 mV. This swing has to be raised at to value of 5 V so it needs a simple non-inverting amplifier with gain of 29.41 which is calculated by:

(2) $$A_{U}=1+\frac{R17_{1-2}}{R17_{2-3}}$$

**Figure 5.**
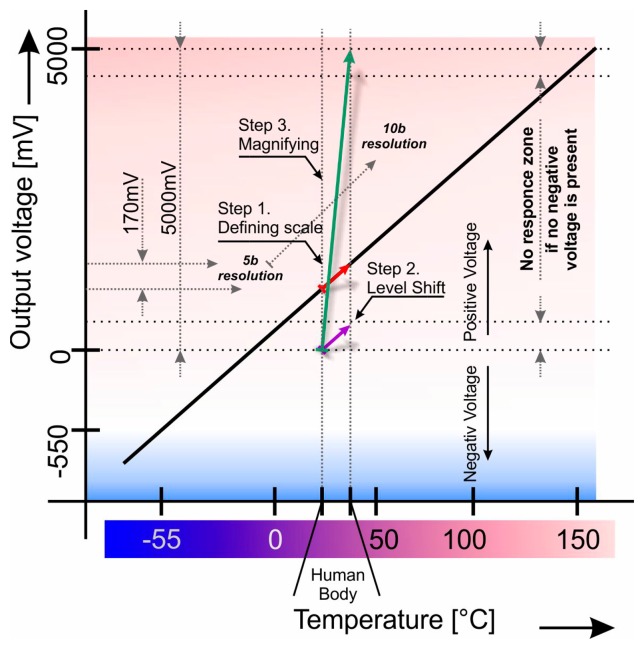
Principle of the presented device.

Considering R17 is a trimmer device, which allows fine tuning of target gain, a 10-turn version is recommended for this case. One more time, the output is computed with a filter to avoid undesired transients and the input signal is amplified by gain value and delivered to the output. This principle mentioned above is schematically represented in Figure 5.

The mechanism we propose is presented In Figure 5. It is used to increase sensitivity when measuring small temperature differences. The original signal that responds to the temperature difference of 17 °C (25 °C–42 °C) is obtained from the LM35 sensor device represented by a red arrow. As seen on the Y axis, 17 °C corresponds to a 170 mV difference, since the LM35 has a sensitivity of 10 mV/°C. The absolute voltage level starts at 0.25 V and ends at 0.42 V yet there is an insignificant difference to convert it to a digital signal, due to the huge conversion error as we mentioned before. It corresponds to a 5 b A/D converter with a full scope coverage and if it is necessary to use all the A/D input range, the input signal has to be shifted down to zero level which corresponds to the violet arrow. That is done by the input stage components in Figure 3. The IC5A with its surrounding resistor network acts as a differential amplifier with selectable reference voltage level (VREF label on resistor R10). Resistor R14 and capacitor C13 act as low pass filters which are optional and serve as filters of any high frequency noise. The output of this stage is a shifted input signal that has the shifted value inverse proportional to the reference voltage level and if it is lowered, the output signal is shifted upwards.

The output stage would only amplify the shifted signal, in order to achieve the full voltage scope to the connected A/D converter represented by a green arrow. The IC5B device is connected as a common non-inverting amplifier with variable gain (trimmer R17) and the output filter (R16 and C16) has the same purpose as mentioned before. The main objective is to achieve full voltage scope from 0 V to 5 V yet this is very complicated, once a standard operational amplifier is used. It has a “no response zone”, therefore it is not possible to reach both extremes—5 V and 0 V—due to the internal components and physical laws. If the amplifier uses a standard complementary transistor, the N-P-N junctions will not allow current to pass through them under 0.6 V and increase the approximate V (Supply voltage) −0.6 V. This state is presented as a dotted line in Figure 5 as “no response zone”. To avoid this problem, we need to use a negative supply voltage which will make the output transistors go into a conduction state, then the output can be as low as 0 V. A similar issue occurs on the opposite voltage level, therefore, one possibility is to increase the supply voltage at least 0.6 V over the 5 V. Higher voltages are not the problem, since another stabilizer could easily be used. On the other hand, the negative voltage issue has to be solved in another way. One solution was presented in Figure 4 before.

## 6. Comparing of Developed Temperature Sensor to Preceding Simulations

**Figure 6.**
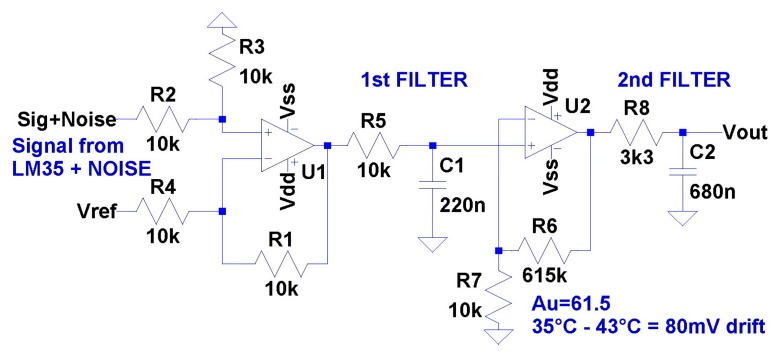
Electric scheme of the temperature sensor realization.

Prior to the realization of the temperature sensor, there is a requirement to perform electric simulations. For this purpose, we used a “LT Spice IV” environment [10] since the first hardware approach would certainly not cause a deadlock. The simulation was based on the following electric scheme in Figure 6 that is developed for real tests and proposed as a functional prototype to be tested for a long period of time inside the incubator.

The electrical scheme in Figure 6 can be divided to four subsections; the first is a differential amplifier, which is the sum of Sig, Noise and Vref consisting of the resistor network (R1 to R4) and the operational amplifier (U1). The second subsection basically consists of a low pass filter (R5 and C1). The third subsection is a non-inverting amplifier (R6, R7, U2) which amplifies the input filtered signal with a fixed gain (Au). Since we are in the low frequency range, the amplifier's gain could be considered as constant. In the case of higher frequency signals, low cost operational amplifiers have higher signal distortion, higher signal phase shift and higher signal edge distortion. All these drawbacks are directly match up with op amp parameters like:

- Slew Rate (0.3 V/μs),
- Bandwidth (700 kHz while Au = 1),
- Open Loop voltage gain and it's frequency dependence.

Generally speaking, the higher values imply the better op amp, nevertheless bring this implies a higher price, however the solution we propose is very cheap. The final price of the temperature sensor is $6 with the PCB, of where the electronic parts cost only $3. The cost is pretty low because of the characteristics of the target system [11–14]. As mentioned above, these temperature systems have very long time constants, so the working frequencies are in order of units of hertz and therefore demands on the quality of electronics parts can be lower. The fourth and final subsection of the electronic scheme is an output low pass filter (R8 and C2) which has a higher damping factor than the first filter.

**Figure 7.**
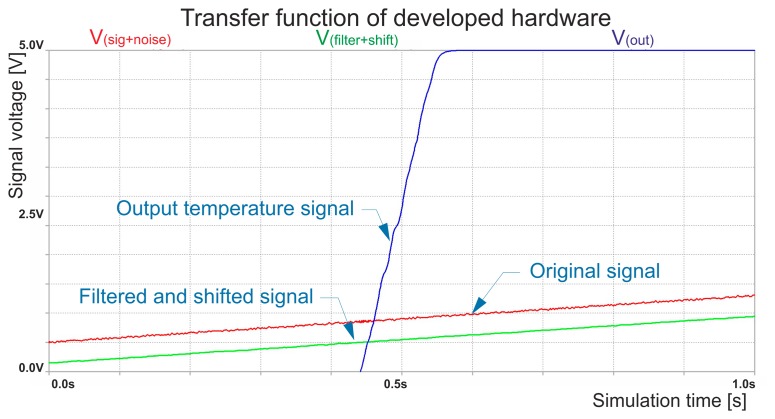
Temperature's sensor transfer function.

There is evidently no way to predict the behavior of a simulated scheme precisely, however in that case the simulated output is very close to the real operational result due to the very low operational system time. Since in general temperature systems comprise very slow processes, there are no rapid, dynamic changes and signal output lies in the static area of operational amplifiers [15]. In Figure 7, the changes in all important signals as they pass through the developed hardware are presented.

The original signal from the LM35 is represented by a red color curve. For test purposes a parasitic noise signal was embedded into the signal element. The red curve is not exactly smooth, since it is deformed by the added noise. The green curve is the shifted as well as filtered original signal as described in Section 5. The final temperature signal is defined by blue color, it has the highest slope and is also passed throughout a simple filter to remove any undesired frequencies. The applied filters also affect the phase of the signal, yet the time shift is negligible.

**Figure 8.**
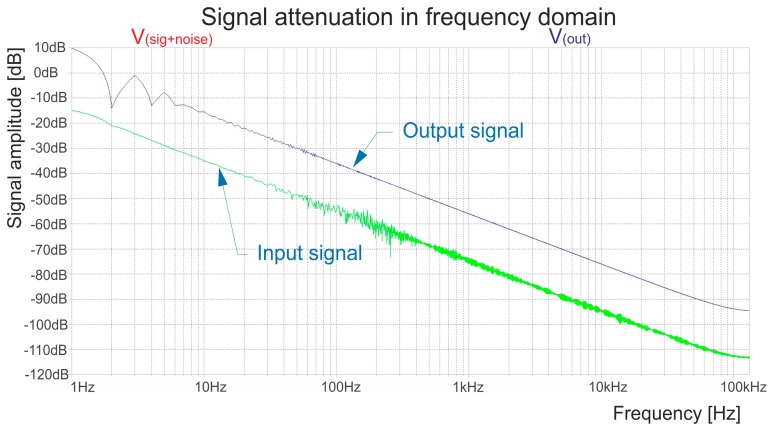
Frequency response of the used filters.

The frequency response is presented in Figure 8, where the input signal (the green curve) is affected by white noise. This simulation proves that the simple filters we used are sufficient and can be used in future hardware designs. The output signal is relatively clean and anything over 100 Hz cannot seriously affect the output signal. The filter design and component value remain the same as in the model. The output signal from the temperature sensor was steady without any undesired spikes and the time delay between the outputs of the LM35 and the hardware design are insignificant.

## 7. Testing of the Developed Solution

The device was tested with a real incubator at a faculty hospital. The target temperature range was from 35 °C to 45 °C which matched the output voltage difference of 170 mV that started at a voltage level of 350 mV. It means that the output voltage fluctuation was from 350 mV to 520 mV which can be seen in Figure 5 as the red arrow. This signal is obtained directly from the temperature sensor and then it leads to the first stage (IC5A on Figure 3). This stage shifts the sensor's voltage down to the zero level which could also be seen in Figure 5 as the shifted voltage represented by the violet arrow. This stage is very important, since this voltage shift means that the zero voltage level presents the minimum measurement temperature (35 °C). The output of this stage is filtered by the R-C network (R14 and C13) and results in the averaging of the output signal which is very important since any stochastic event is attenuated and the R-C network has to be properly set. The presented values are not critical, because the human body temperature changes very slowly. Mathematically, this network can be described by:

(3) $$V_{\text{out mean}}=\frac{1}{T}\cdot \int_{0}^{T}v_{\text{out}}\left(t\right)\cdot dt$$

where ***V_out-mean_*** represents the mean value in the ***T*** time interval and ***v_out_*(*t*)** is the actual voltage before processing in time ***t***. Any input anomalies which differ from the mean voltage value are converted into energy increments (decrements) at the filtration capacitor (C13), which could be approximated by:

(4) $$\Delta E_{t1∼t2}=\frac{C}{2R}\cdot \int_{t1}^{t2}{\left(v_{\text{out}}\left(t\right)-V_{\text{out}-\text{mean}}\right)}^{2}\cdot dt$$

It can be easily assumed that any input voltage spike which emerges from time ***t*1** until ***t*2** will lead to an energy increment in the capacitor. This network also proceeds to transient response on op amp.

The second stage, which corresponds IC5B in Figure 3, only amplifies the modified signal. The working principle is represented in Figure 5 by the green arrow. Voltage gain ***Au*** is 29 which means that voltage difference of 170 mV multiplied by 29 equals about 5 V. This voltage then covers the full range of connected A/D and its full scope can be utilized. The final output voltage is also filtered by the same type of R-C network to avoid any undesired output voltage spikes.

**Figure 9.**
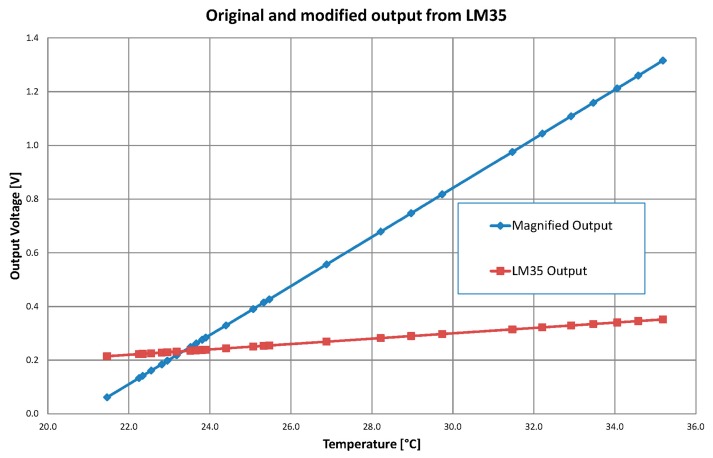
Calibration measurement with real system voltage outputs.

A number of real measurements was conducted and the basic testing of the device was simple. While increasing system's temperature, the output voltage on the LM35 and on the device's output were monitored. The LM35 voltage output should generate a voltage at a level that can be expressed by multiplying the input temperature by 10 mV which is represented by the red curve in Figure 9. Therefore the magnified voltage which is the blue curve in Figure 9 was moved down and the slope was increased and thus the resulting sensitivity became higher. The original LM35 output can be mathematically derived as *y* = 0.01*x*, while the modified, magnified device's output can be described as *y* = 0.0914*x* − 1.8995. Given the expressions, it can be assumed that the voltage gain was set to the value 9.14 and a −1.8995 V level shift was applied.

Additionally, the calibration measurement with a referential temperature sensor is presented in Table 2. The purpose of this measurement is to verify whether the proposed device is working properly. The temperature slowly rises from its minimum level of 21.5 °C until it reaches 35.3 °C. From Figure 9 it could be seen that the output signal (blue curve) is moved down by 0.153 V and magnified with gain A_u_ = 9.14^±0.01^. The original output from the LM35 temperature sensor is represented by the red curve. From the graph it is evident that the device is working properly. The original voltage drift from the LME sensor is 0.137 V while the drift from the device is 1.25 V.

The real temperature was measured on an accurate measuring device—an ALMEMO 2290-8. Data from this device were considered as reference points and this particular referential device was used since it has high accuracy of 0.05%. The output information can be used as a reference in this particular measurement. The ALMEMO 2290-8 is a specialized data logger with thermocouple inputs and with the ability to store 100,000 measurement datapoints.

## 8. Measuring Temperature Profiles in the Incubator

**Figure 10.**
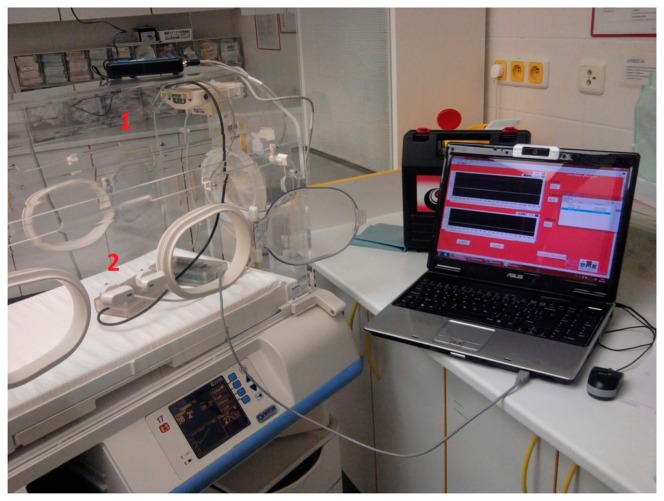
Testing environment inside a real Dräger 8000SC incubator [16]. The developed temperature sensor is mounted on the top of incubator in the same position as the original one (indicated as number **1**). There is also a second place **2** where the developed sensor is placed mounted on the same place where a newborn baby is placed when incubator is in use.

One of the main issues of this project was the comparison of the developed solution with the original one. Control measurements inside the incubator [16] were performed at the University Hospital Ostrava and the measuring device was placed inside an incubator instead of a baby. Our first temperature sensor was located in the same place as a single integrated temperature sensor of the original incubator which can be seen in Figure 10. Additionally, the measured data are shown in Table 3 below.

Table 4 shows the differences in measured temperatures; the first column shows testing temperatures *vs.* original. The second column shows the calculated temperature differences between the original (set) temperatures and those measured at the top of incubator—the temperature at position 1. The third column shows the temperature difference measured at the top (positon 1) and bottom (position 2—instead of a baby). During the testing process of the developed solution inside the incubator, our results showed that the temperature set on the incubator is sensed only at the top of incubator, where the deviation of the output from our developed sensor is 0.1 to 1.4 °C which is not a very high difference. Next case however is more astonishing given that the newborn baby bed temperature is significantly lower. The difference of the actual temperature varied in the range from 2.0 to 5.3 °C in Table 3, particularly in the range of temperature values from 36 to 37 ° C, which are the most frequently used temperatures. In addition the temperature difference was almost 4 °C, which is a significant difference for the comfort of a newborn baby.

### 8.1. Testing of the Temperature Sensor Response Time

We additionally focused on another test concerning the response time of the new temperature sensor. The sensor was mounted in the package in which it will be used and in a box which routed to the cable and it was gradually heated by a hot-air blower from 24 °C to 40 °C and then cooled spontaneously to room temperature. The time between changes of both temperatures were recorded in 1 °C steps, which can be seen in Tables 5 and 6.

From the results it is noticeable that the new sensor displays a very fast response time when the temperature changes, especially when it is increasing. It gives the value in the desired range after no more than 1 min, however spontaneous cooling times were significantly longer for both sensors. The internal sensor is slightly handicapped by being enclosed in boxes, nonetheless it has ability to measure temperature changes in relatively short periods of time such as 10 s.

These tables can serve as a tool for estimating the time required for analyzing the actual functionality of the incubators. Generally a time of 10 min for increasing temperatures and 30 min for decreasing temperatures can be recommended to get a required temperature to be set correctly.

## 9. Discussion

Initially, the introduced method was developed to be used in neonatal incubator devices. In these devices it is very important to measure temperature as precise as possible since the life of the babies depends on it. The mentioned procedure use a hardware magnifier which is able to increase the final temperature resolution and utilizes the full range of the connected A/D converter. Other methods mentioned in the related work section are either too complex, expensive or rigid to be implemented as default accessories. The achieved accuracy is sufficient to be a primary data acquisition source when controlling target temperatures in an incubator.

The proposed electronic circuit has very good linearity, since no inductor or capacitive parts are used in the feedback loop. Therefore, the only nonlinearity that emerged is the thermal float of the resistors' resistance, which can be considered as zero for the temperature range and in the fluctuation used in a medical environment. This is only an instrument in the subsequent control loop, which is responsible for the precise temperature reading. Other techniques mostly describe whole thermal control solution, which may not be useful in all conditions.

From the obtained measurement results, the final op amp gain was set to cover the whole A/D input range which is the first of the two necessary setups and the second setup is concerned with the voltage shift which responds to particular sensor characteristics. The versatility of the design is the key feature since with minimum changes, it can be used to increase the measurement sensitivity of relative air humidity, to optimize output swinge when measuring air flow in the incubator, when increasing the dynamic range of a light sensor device and so on.

## 10. Conclusions

This paper basically focuses on the problems of temperature sensors when they have to be connected to an A/D converter. Almost in all the cases the output of the sensor is in a “default” state without any signal adjustments. This leads to decreasing sensitivity of the sensors and consequently to wasting A/D capabilities. One of the typical problems is the measurement of temperatures in prenatal incubators. There is a small range of temperatures, which has to be kept at a stable level according to the needs of the patient. If it is necessary to measure a narrow range of temperatures with a sensor which has a bigger temperature range, the total temperature measurement accuracy will drastically drop, due to small voltage drifts of the sensor's output.

The proposed matching network will ensure that small temperature/voltage drifts will be properly matched to the connected A/D and the final accuracy reading will be as high as possible. The presented matching network can be considered as a hardware voltage magnifier/shifter, therefore we were able to use the full output voltage swing without any limitations, such as dead zones on op amps border voltage levels. The developed electronic circuit is easily integrated as a default system into the temperature control loop in the hospital environment, especially in prenatal incubator control. This could help stabilize the incubator's inner temperature more precisely and maximize its effects on the patient.

## Figures and Tables

**Table 1.** Comparison of the existing method for precise temperature measuring.

**Article/Method**	**Temperature Measurement Accuracy**	**Method Complexity**	**Price**	**Versatility**
NMOS temp. dependence [7]	0.55 °C	Moderate	Moderate	Low
Sound speed method [8]	0.2 °C	Very High	High	Low
Pt-100 and high precision thermometer [9]	0.02 °C	High	Very High	Low
Our solution	0.2 °C or better	Very Low	Very Low	High

**Table 2.** Measured data from the device's output, LM35 output and referential device.

**Real Tmp**	**LM35 Tmp**	**A/D**	Device **Output**	**LM35 Output**
21.5 °C	21.5 °C	50	0.061035 V	0.214600 V
22.3 °C	22.3 °C	109	0.133057 V	0.222500 V
22.4 °C	22.3 °C	116	0.141602 V	0.223400 V
22.6 °C	22.6 °C	132	0.161133 V	0.225500 V
22.9 °C	22.8 °C	151	0.184326 V	0.228100 V
23.0 °C	23.0 °C	162	0.197754 V	0.229500 V
23.2 °C	23.2 °C	179	0.218506 V	0.231800 V
23.5 °C	23.5 °C	204	0.249023 V	0.235200 V
23.7 °C	23.7 °C	215	0.262451 V	0.236600 V
23.9 °C	23.8 °C	226	0.275879 V	0.238100 V
23.9 °C	23.9 °C	233	0.284424 V	0.239000 V
24.3 °C	24.4 °C	270	0.329590 V	0.244000 V
25.0 °C	25.1 °C	320	0.390625 V	0.250700 V
25.3 °C	25.3 °C	340	0.415039 V	0.253300 V
25.5 °C	25.5 °C	350	0.427246 V	0.254700 V
26.8 °C	26.9 °C	456	0.556641 V	0.268800 V
28.0 °C	28.2 °C	556	0.678711 V	0.282200 V
28.7 °C	29.0 °C	612	0.747070 V	0.289700 V
29.5 °C	29.7 °C	670	0.817871 V	0.297400 V
31.7 °C	31.5 °C	799	0.975342 V	0.314700 V
32.2 °C	32.2 °C	855	1.043701 V	0.322100 V
32.9 °C	32.9 °C	908	1.108398 V	0.329200 V
33.6 °C	33.5 °C	949	1.158447 V	0.334700 V
34.1 °C	34.1 °C	993	1.212158 V	0.340600 V
34.6 °C	34.6 °C	1032	1.259766 V	0.345800 V
35.3 °C	35.2 °C	1078	1.315918 V	0.351900 V

**Table 3.** Measured temperatures inside the incubator.

**Temperature Setting on the Incubator [°C]**	**Temperature 2 [°C]**	**Temperature 1 [°C]**
32	29.55	32.12
33	31.47	33.50
34	32.21	34.31
35	32.92	36.14
36	33.47	37.07
37	34.06	38.02
38	34.58	39.33
39	35.19	40.44

**Table 4.** Difference in measured temperatures inside the incubator.

**Temperature in Incubator [°C]**	**Δ (t_orig_ − t_1_) [°C]**	**Δ (t_1_ − t_2_) [°C]**
32	0.12	2.57
33	0.5	2.03
34	0.31	2.1
35	1.14	3.22
**36**	**1.07**	**3.6**
**37**	**1.02**	**3.96**
38	1.33	4.75
39	1.44	5.25

**Table 5.** Time delay of the sensors when the temperature is increasing.

**Temperature [°C]**	**Time of Original Sensor [mm:ss]**	**Time of New Sensor [mm:ss]**
24	0:00	0:00
25	1:04	0:04
26	1:45	0:07
27	2:20	0:15
28	2:30	0:16
29	2:38	0:17
30	2:51	0:18
31	3:08	0:19
32	3:28	0:20
33	3:49	0:21
34	4:15	0:23
35	4:45	0:25
36	5:16	0:26
37	5:50	0:29
38	6:33	0:32
39	7:16	0:37
40	7:52	0:41

**Table 6.** Time delay of the sensors when the temperature is decreasing.

**Temperature [°C]**	**Time of Original Sensor [mm:ss]**	**Time of New Sensor [mm:ss]**
40	0:00	0:00
39	1:57	0:16
38	3:37	0:20
37	4:47	0:34
36	5:30	0:47
35	6:35	1:03
34	7:26	1:19
33	9:15	1:38
32	10:20	2:03
31	11:36	2:31
30	13:17	3:05
29	15:50	3:45
28	18:14	4:31
27	19:50	5:30
26	22:10	7:09
25	27:28	10:08
